# Does the Summer Season Affect the Amniotic Fluid Volume during Pregnancy?

**DOI:** 10.3390/ijerph18189483

**Published:** 2021-09-08

**Authors:** Ah-Young Choi, Jun-Yi Lee, In-Sook Sohn, Han-Sung Kwon, Yong-Soo Seo, Myoung-Hwan Kim, Seung-Woo Yang, Han-Sung Hwang

**Affiliations:** 1 Division of Maternal and Fetal Medicine, Department of Obstetrics and Gynecology, Research Institute of Medical Science, Konkuk University School of Medicine, Seoul 05030, Korea; 20180079@kuh.ac.kr (A.-Y.C.); 19960011@kuh.ac.kr (I.-S.S.); 20050024@kuh.ac.kr (H.-S.K.); 2 Department of Obstetrics and Gynecology, Korea University Guro Hospital, Korea University School of Medicine, Seoul 08308, Korea; junielee99@gmail.com; 3 Department of Obstetrics and Gynecology, Sang-Gye Paik Hospital, Inje University School of Medicine, Seoul 01757, Korea; obdrseo@paik.ac.kr (Y.-S.S.); myankim@paik.ac.kr (M.-H.K.)

**Keywords:** amniotic fluid volume, oligohydramnios, summer season

## Abstract

Amniotic fluid is crucial for the well-being of the fetus. Recent studies suggest that dehydration in a pregnant woman leads to oligohydramnios. We assessed the variation in the amniotic fluid index (AFI) during the summer and non-summer seasons and evaluated neonatal outcomes. We retrospectively reviewed electrical medical records of pregnant women who visited the Konkuk University Medical Center for antenatal care, between July 2005 and July 2019. A total of 19,724 cases from 6438 singleton pregnant women were included after excluding unsuitable cases. All AFI values were classified as 2nd and 3rd trimester values. Additionally, borderline oligohydramnios (AFI, 5–8) and normal AFI (AFI, 8–24) were assessed according to the seasons. The average AFI between the summer and non-summer season was statistically different only in the 3rd trimester; but the results were not clinically significant. In the 3rd trimester, the summer season influenced the increased incidence of borderline oligohydramnios. The borderline oligohydramnios group showed an increased small-for-gestational-age (SGA) rate and NICU admission rate. In the summer season, the incidence of borderline oligohydramnios was seen to increase. This result would be significant for both physicians and pregnant women.

## 1. Introduction

The amniotic fluid was previously considered nonfunctional; however, it is now well known that the amniotic fluid is crucial for the lung maturation, gastrointestinal tract development, and neuromuscular development of the fetus. Approximately 98% of the amniotic fluid comprises water. The amniotic fluid volume increases from around 30 mL at 10 weeks of gestation to 800 mL during the mid-third trimester [1,2]. The amniotic fluid volume is regulated by four main pathways: (i) fetal urination, (ii) intramembranous osmolality difference, (iii) respiratory tract, and (iv) fetal swallowing. An abnormality in the amniotic fluid volume may result from fetal or placental pathology [3]. The amniotic fluid index (AFI) has been an integral component of fetal assessment during an antepartum ultrasound examination for more than 20 years [1,4,5]. Generally, AFI is calculated by measuring the sum of the vertical depths of the largest pocket in each quadrant or a single deepest pocket [6]. The AFI is generally considered normal if it is greater than 5 cm and less than 24 cm. A decrease in amniotic fluid, oligohydramnios, is typically defined as an AFI of less than 5 cm, which represents a value below the first percentile [7]. A borderline AFI has been defined by different authors who report different cut-off values. Phelan et al. defined borderline oligohydramnios as an AFI of 5–8 cm [8,9,10]. While Gumus defined borderline oligohydramnios as an AFI of 5–10 cm [11], Kreiser defined it as an AFI of greater than 5 cm, but below the 2.5 percentile. In the present study, considering that normal AFI is 5–24 cm, AFI values between 5 cm and 8 cm were classified as “borderline oligohydramnios” [5].

According to previous studies, there are five etiologies of oligohydramnios, including fetal, placental, maternal, pharmacologic, and idiopathic etiologies [12]. Fetal and placental abnormalities are often associated with fetal growth restriction. The underlying cause in such cases is placental insufficiency or fetal urinary tract anomalies [12,13]. Maternal factors, such as preeclampsia or vascular disease, are also associated with oligohydramnios. Oligohydramnios has been found to be associated with non-steroid anti-inflammatory drugs (NSAIDs) and angiotensin converting enzyme (ACE) inhibitors [14]. Oligohydramnios is also associated with adverse pregnancy outcomes and with a high risk of fetal malformations, stillbirth, growth restriction, non-reassuring heart rate pattern, and meconium aspiration syndrome. Additionally, fetal malpresentation problems and umbilical cord compression are easily observed in oligohydramnios [10]. The risk for cesarean delivery due to fetal distress and the risk of an APGAR score < 7 at 5 min are higher during oligohydramnios than in pregnancies with a normal AFI [11,15,16,17,18]. Thus, measuring the amniotic fluid volume is important for fetal assessment during pregnancy and, in recent years, strategies to increase the amniotic fluid volume have gained much attention [13,19].

Oligohydramnios, without any evidence of either maternal or fetal abnormalities, is known as isolated oligohydramnios [20]. In the literature, the association between idiopathic oligohydramnios and hot weather remains controversial. Luton et al. observed a substantially higher incidence of oligohydramnios during a Paris heat wave [21]. Some researchers claim that the AFI correlates with the maternal hydration status [19]. However, maternal fasting shows no significant correlation with AFI [22]. Water consumption and clinical dehydration are known to commonly occur in June, July, and August, in the northern hemisphere. Since South Korea is also located in the northern hemisphere, and its summers seasons are hot and humid, maternal dehydration could be a common occurrence in South Korea [23]. Some studies have assessed the effectiveness of maternal hydration in increasing amniotic fluid volume, and reported that maternal hydration and intravenous hypotonic hydration are associated with an increase in the amniotic fluid volume [13,24]. Yet, studies on amniotic fluid and its seasonal variation are rare, especially in the Korean population.

This study was designed to determine whether the summer season is a risk factor for oligohydramnios, by comparing the frequency of oligohydramnios during the summer months and that during the other months, in South Korea. The secondary outcome was to compare and interpret the neonatal outcomes of the two seasonal groups

## 2. Materials and Methods

### 2.1. Patients

**Figure 1:**
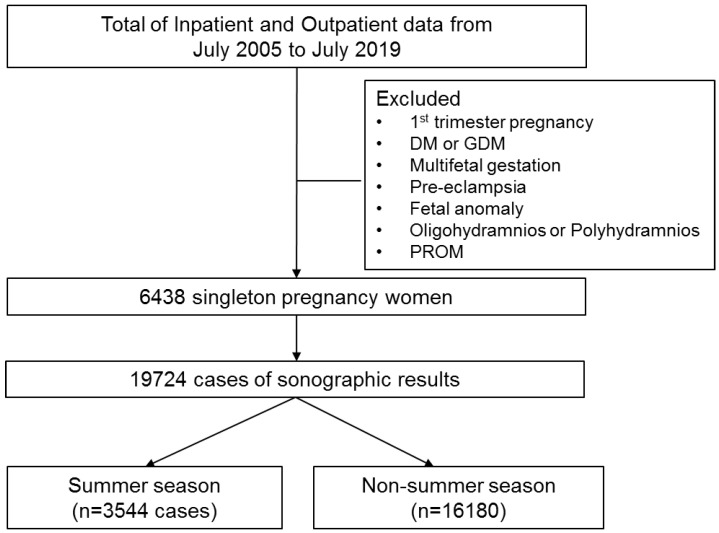
Data of patient enrollment.

A retrospective study was performed in Konkuk University Hospital from July 2005 to July 2019. The data of all medical records of pregnant women were reviewed. We excluded the data of pregnant women in the 1st trimester pregnancy, and with oligohydramnios (AFI, <5), polyhydramnios (AFI, >24), premature rupture of membrane (PROM), diabetes mellitus (DM), gestational diabetes mellitus (GDM), pre-eclampsia, multifetal gestation, and fetal anomaly that causes abnormal amniotic fluid volume. A total of 6438 singleton pregnant women with 19,724 cases of AFI were evaluated. Further, all the 19,724 cases were classified by the season of measurement (summer season, 3544 cases; non-summer season, 16,180 cases; Figure 1). Maternal demographic and obstetric characteristics were evaluated. Regarding to neonatal outcome, gestational age at birth, birth weight, 1 min APGAR score, 5 min APGAR score, the risk of small for gestational age (SGA), and the rate of neonate intensive care unit (NICU) admission were analyzed for borderline oligohydramnios. SGA was defined as a weight below the 10th percentile of the gestational age [25]. Other neonatal outcomes, such as respiratory distress syndrome (RDS), meconium aspiration syndrome (MAS), neonatal jaundice, and neonatal death, were reviewed through the electronic medical records throughout the study period. RDS was defined as an infant with neonatal respiratory distress syndrome often being born premature and presenting with signs of respiratory distress usually immediately after delivery, or within minutes of birth [26]. MAS was defined as any respiratory distress occurring soon after birth in an infant born from a meconium-stained amniotic fluid with compatible chest radiologic findings [27].

### 2.2. Measuring AFI According to the Seasons

The seasons were divided into summer and non-summer. The summer season comprised the months of July and August, while the non-summer season comprised the remaining months. The summer and non-summer season were compared with monthly average temperature and humidity data from the Korea Metrological Administration from 2005 to 2019. Additionally, borderline AFI (AFI, 5–8) and normal AFI (AFI, 8–24) were evaluated according to the seasons [23]. To measure the amniotic fluid volume, standard ultrasound examination was performed by medical faculties, those who had been working in the same medical center. The AFI was calculated as the sum of the deepest vertical pocket of the four quadrants. The patient laid down in supine position, and the uterus was randomly divided into four quadrants using linea nigra and umbilicus. AFI values were sorted by trimesters, i.e., the 2nd and 3rd trimesters [5,7,28]

### 2.3. Statistical Analysis

Data were analyzed using the statistical software package SPSS (version 18.0; SPSS Inc., Chicago, IL, USA). Median values were used to describe continuous data, with discrete variables displayed as totals and frequencies. For univariate analyses, Mann–Whitney U test was used to compare continuous data. Chi-squared test or Fisher’s exact test were used for categorical variables, as appropriate. The Pearson correlation coefficient was used to determine the association between AFI and humidity or temperature. The Chi-squared test was used to calculate the odds ratio of the summer season affecting borderline oligohydramnios.

## 3. Results

**Figure 2:**
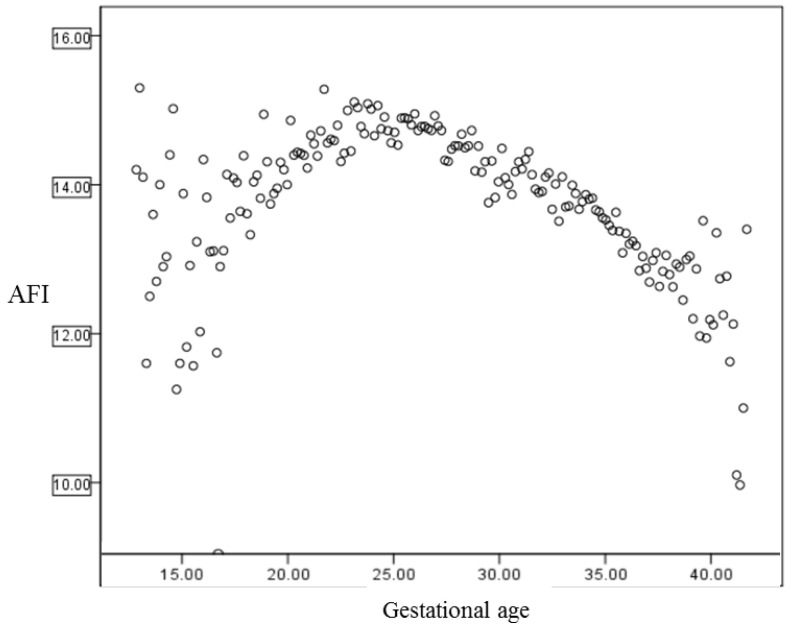
Average AFI throughout each gestational age.

During the study period, a total of 19,724 cases were evaluated. Figure 2 shows the average AFI of each gestational age during the 2nd and 3rd trimesters. Similar to previous studies, the average AFI increased throughout the 2nd trimester and slightly decreased in the 3rd trimester [1,29].

Maternal characteristics of the study participants are shown in Table 1.

The average AFI, temperature, and humidity during the study period (2005–2019) were compared between the summer (July and August) and the non-summer season (Table 2). During the 2nd trimester, the average AFI in the summer season was 14.6 (n = 900 cases) and in the non-summer season was 14.6 (n = 4296 cases). In the 3rd trimester, the average AFI in the summer season was lower than that in the non-summer season (13.5 of 2644 cases versus 13.7 of 11,884 cases, *p* = 0.005). The average temperature and humidity were different between the summer and non-summer seasons (2nd trimester: summer, 25.9 °C and 57.1%; non-summer, 10.2 °C and 72.9%; 3rd trimester: summer, 25.8 °C and 72.9%; non-summer, 10.1 °C and 57.1%).

In Table 3, humidity and temperature are shown to be highly correlated in this study (2nd trimester: *r* = 0.815, *p* < 0.001; 3rd trimester: *r* = 0.819, *p* < 0.001). However, AFI was neither correlated with humidity nor temperature in the entire study period.

Although the AFI did not show any seasonal differences, we evaluated borderline oligohydramnios in the same population (Table 4). The percentage of borderline oligohydramnios cases was not significantly different between the seasons. However, in the 3rd trimester, the number of cases with borderline oligohydramnios was 268 (10.1%) in the summer and 966 (8.1%) in the non-summer seasons. Moreover, in the 3rd trimester, the summer season had an influence on the increased incidence of borderline oligohydramnios than did the non-summer season (Odds ratio: 1.275, 95% CI: 1.106–1.470, *p* < 0.001).

To compare neonatal outcomes, the data were compared between the normal AFI and borderline oligohydramnios pregnancy groups in the 3rd trimester (Table 5). The median gestational age and APGAR score were not different between the groups. However, a difference in the median birth weight was observed between the groups: 3365 g in the normal AFI group and 3080 g in the borderline oligohydramnios group (*p* < 0.001). Additionally, SGA and NICU admission rates were higher in the borderline oligohydramnios group than in the normal AFI group (6.6% vs. 22.6%, *p* < 0.001; 5.2% vs. 7.9%, *p* = 0.016, respectively). Neonatal outcomes, including RDS, MAS, and neonatal jaundice, showed no significant differences between the two groups.

## 4. Discussion

AFI is a used factor for predicting the neonatal outcome; it is also believed to be associated with the weather. Previous studies found that maternal dehydration is more common in the summer season, thereby increasing the risk for oligohydramnios in the summer season. This study examined whether the summer season influences the AFI in normal pregnant women. In this study, the average AFI between the summer and non-summer season was statistically different in only the 3rd trimester (13.5 cm vs. 13.7 cm, *p* = 0.005), which was clinically insignificant. However, the risk for borderline oligohydramnios was observed to be higher in the summer season.

A previous study revealed that oligohydramnios was significantly more common during the summer months than during the other months [30]. Luton et al. published a study on the Paris heat wave and the occurrence of oligohydramnios and revealed that hot weather may result in oligohydramnios [21]. Previous studies have also shown that in dehydrated conditions, there is a general water loss that may influence the amniotic fluid volume [13,19]. Data from the Korea Meteorological Administration showed that the summer season in Korea is hot and humid, and we could assume that clinical dehydration is more common during the summer than it is in other seasons [23]. In this study, however, no significant change in the AFI during the summer season was observed, but the incidence of borderline oligohydramnios was higher during the summer season in women in the 3rd trimester.

An AFI of <5 cm is a meaningful cut-off value for predicting adverse pregnancy outcomes. AFI values between 5 cm and 8 cm were termed ‘borderline’ by Moore and colleagues [1]. Gumus found that pregnancy with borderline AFI values had a higher risk for preterm birth, fetal distress during labor, and intrauterine fetal growth restriction [11]. Petrozella et al. conducted a large cohort study comparing the normal AFI group and the borderline oligohydramnios group [10]. Similar to Petrozella’s study, borderline oligohydramnios was considered problematic for clinical decision making, and was considered as a normal AFI volume, in this study. Maternal characteristics, the induction of labor, and mode of delivery were almost similar in both groups, indicating that the clinical intervention was not different despite the borderline oligohydramnios.

Borderline oligohydramnios is a recent concept, and studies performed on this topic are very few. The term “borderline oligohydramnios” is defined in several definitions. Generally, an AFI value between 5 cm and 8 cm is considered as borderline oligohydramnios [11,26,28]. Gumus et al. defined borderline oligohydramnios as an AFI between 5 cm and 10 cm [11]. Borderline oligohydramnios increases the risk for preterm birth, fetal distress, and an increase in the rate of SGA [12,31,32,33]. Petrozella et al. found that in preterm pregnancy, the risk of fetal malformation is 10-fold higher in the oligohydramnios group and 5-fold higher in the borderline oligohydramnios group than that found in the normal AFI group [10]. An AFI value of <8 cm is shown to increase the rate of preterm birth. Additionally, the prevalence of fetal growth restriction was nine-fold higher in the oligohydramnios group and five-fold higher in the borderline oligohydramnios group than in the normal AFI group. Furthermore, when diagnosed at term, the APGAR score was lower in the borderline oligohydramnios group than in the normal AFI group [8,11]. The neonatal outcome of the present study showed similar results [33].

As mentioned above, the concept of borderline oligohydramnios has been important recently. However, the study of idiopathic borderline oligohydramnios is less common. The seasonal variation of borderline oligohydramnios is rarer. Even though maternal dehydration, which reflects a decreased amniotic fluid volume, is well known, research on the relationship between the weather and amniotic fluid volume is scarce [3,8,13,22]. Previous studies have mainly focused on abnormal AFI and oligohydramnios. In this study, the risk for borderline oligohydramnios was higher during the summer season, which was indicated by the neonatal outcome data. The median birth weight was lower, and the rate of SGA was higher in the borderline oligohydramnios group. NICU admission was also more common in the borderline oligohydramnios group than in the normal AFI group.

This study had several limitations. First, this study was retrospective and some factors might have affected the AFI. For instance, personal habits such as the water consumption status were not well controlled. Other maternal confounding factors also exist, including gestational age at diagnosis, the presence of preterm labor, and other maternal diseases. Regarding neonatal outcome, the SGA rate was higher in the borderline oligohydramnios group than in the other group. Therefore, although there were no differences in the gestational age at birth, various complex phenomena affect the SGA in borderline oligohydramnios, and a clinical understanding of this should be considered and studied further. The strength of this study was that it was a single-center study with the same sonographic measurements and with the same clinical treatment. The method of measuring the AFI was not largely different among the clinicians. Since a longitudinal follow-up of the same patient was done, the bias in inter-patient data was very low. Other major factors such as ethnicity, religion, and socio-economic status were not different. Additionally, this study focused on borderline oligohydramnios, which is still generally considered as a normal AFI. As there is few research on the seasonal variation of borderline oligohydramnios, these findings provide indications for clinical counseling. Therefore, borderline oligohydramnios should be closely observed and managed for better neonatal outcomes, especially in the Korean population [20].

In conclusion, the summer season in Korea is hot and humid, and this may cause dehydration in pregnant women. Although the average AFI during the summer and non-summer seasons was statistically different, it was not significant to make clinical decisions. However, the incidence of borderline oligohydramnios was higher in the summer season in the 3rd trimester of pregnancy, which was similar to other previous studies, that have reported that oligohydramnios is more common in the summer season. Generally, borderline oligohydramnios is still considered as a normal AFI; however, we found that the neonatal outcome in the borderline oligohydramnios group was significantly different from that in the normal AFI group. Thus, it is important for both clinicians and patients to consider the incidence of borderline oligohydramnios, especially during the summer season in Korea.

## 5. Conclusions

In the 3rd trimester, the summer season influenced the increased incidence of borderline oligohydramnios. The borderline oligohydramnios group showed an increased SGA rate and NICU admission rate than those shown by the other group. In the summer season, the incidence of borderline oligohydramnios was observed to have increased. These results would be significant for both physicians and pregnant women.

## Figures and Tables

**Table 1:** Clinical characteristics of the study population.

Characteristics	Value
Maternal age	32.0 (21.0–42.0)
Gestational age at birth	39.0 (32.0–41.3)
BMI at birth	26.4(20.4–36.5)
Parity	
Nulliparity	3374 (52.4%)
Multiparity	3064 (47.6%)
Labor induction	
No labor induction	4009 (62.3%)
Labor induction	2429 (37.7%)
Delivery	
Vaginal delivery	4141 (64.3%)
Cesarean delivery	3576 (55.5%)

Data are presented as median (range) or number (percentages) values.

**Table 2:** Amniotic fluid index (AFI), temperature, and humidity in the summer versus non-summer seasons for a period of 15 years.

	Summer	Non-Summer	*p* Value
AFI in 2nd trimester	14.6 ± 2.3	14.6 ± 2.4	0.727
AFI in 3rd trimester	13.5 ± 2.9	13.7 ± 2.9	0.005
Temperature (°C)	25.9 ± 9.1	10.2 ± 1.1	<0.001
Humidity (%)	72.9 ± 4.1	57.1 ± 2.0	<0.001

Data are presented as mean ± SD. Analysis was by Mann–Whitney U test. A *p*-value of < 0.05 with a 95% confidence interval was considered significant.

**Table 3:** Correlation among AFI, temperature, and humidity in the 2nd and 3rd trimester of pregnancy.

	2nd Trimester				3rd Trimester			
	Humidity (%)		Temperature (°C)		Humidity (%)		Temperature (°C)	
	*r*	*p*	*r*	*p*	*r*	*p*	*r*	*p*
AFI (cm)	−0.001	0.952	−0.001	0.980	−0.16	0.056	−0.09	0.265
Temperature (°C)	0.815	<0.001			0.819	<0.001		

A *p*-value of <0.05 with a 95% confidence interval was considered significant. An *r* indicates the Pearson correlation coefficient.

**Table 4:** Risk of borderline oligohydramnios in summer versus non-summer seasons on 2nd and 3rd trimester of pregnancy.

	Summer	Non-Summer	*p*	OR (95% CI)
Borderline oligohydramnios in 2nd Trimester	21 (2.3)	62(1.4)	0.530	1.63 (0.99–2.69)
Borderline oligohydramnios in 3rd Trimester	268 (10.1)	966 (8.1)	<0.001	1.27 (1.11–1.47)

Data: case number (%). Abbreviation: OR, odds ratio; CI, confidence interval.

**Table 5:** Comparison of neonatal outcomes between normal AFI and borderline oligohydramnios in the 3rd trimester.

	Normal AFI (n = 2326)	Borderline Oligohydramnios, (n = 226)	*p* Value
Gestational age at birth	39.0 (32.0–41.3)	39.1 (32.4–41.3)	0.846
Birth weight	3365.0 (1576–4580)	3080.0 (1465–3890)	<0.001
1 min APGAR score	8 (7–9)	8 (6–8)	0.867
5 min APGAR score	9 (8–10)	9 (8–9)	0.584
SGA	153 (6.6%)	51 (22.6%)	<0.001
NICU admission	121 (5.2%)	18 (7.9%)	0.016
RDS	37 (1.6%)	9 (4.1%)	0.481
MAS	74 (3.2%)	9 (4.1%)	0.831
Neonatal jaundice	563 (24.2%)	38 (16.8%)	0.450
Neonatal death	None	None	

The gestational age, birth weight, and APGAR score are presented as median (range). SGA, NICU admission, RDS, MAS, and neonatal death are presented as number (percentage). Analysis was by Mann–Whitney U test and Fisher’s exact test. A *p*-value of <0.05 with a 95% confidence interval was considered significant. *Abbreviation*: SGA, small for gestational age; NICU, neonatal intensive care unit; RDS, respiratory distress syndrome; MAS, meconium aspiration syndrome.

## Data Availability

The data presented in this study are openly available.
